# Hop stunt viroid: A polyphagous pathogenic RNA that has shed light on viroid–host interactions

**DOI:** 10.1111/mpp.13022

**Published:** 2020-12-10

**Authors:** Joan Marquez‐Molins, Gustavo Gomez, Vicente Pallas

**Affiliations:** ^1^ Institute for Integrative Systems Biology (I2SysBio) Consejo Superior de Investigaciones Científicas, Universitat de València Paterna Spain; ^2^ Instituto de Biología Molecular y Celular de Plantas Consejo Superior de Investigaciones Científicas, Universitat Politècnica de València Valencia Spain

**Keywords:** epigenetics, movement, pathogenesis, replication, viroids

## Abstract

**Taxonomy:**

*Hop stunt viroid* (HSVd) is the type species of the genus *Hostuviroid* (family *Pospiviroidae*). The other species of this genus is *Dahlia latent viroid*, which presents an identical central conserved region (CCR) but lacks other structural hallmarks present in *Hop stunt viroid*. HSVd replication occurs in the nucleus through an asymmetric rolling‐circle model as in the other members of the family *Pospiviroidae*, which also includes the genera *Pospiviroid*, *Cocadviroid, Apscaviroid*, and *Coleoviroid*.

**Physical properties:**

*Hop stunt viroid* consists of a single‐stranded, circular RNA of 295–303 nucleotides depending on isolates and sequence variants. The most stable secondary structure is a rod‐like or quasi‐rod‐like conformation with two characteristic domains: a CCR and a terminal conserved hairpin similar to that of cocadviroids. HSVd lacks a terminal conserved region.

**Hosts and symptoms:**

HSVd infects a very broad range of natural hosts and has been reported to be the causal agent of five different diseases (citrus cachexia, cucumber pale fruit, peach and plum apple apricot distortion, and hop stunt). It is distributed worldwide.

**Transmission:**

HSVd is transmitted mechanically and by seed.

## Graphical abstract

This pathogen profile summarizes the current knowledge on *Hop stunt viroid*, a polyphagous pathogenic RNA that has shed light on significant aspects of viroid–host interactions.

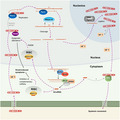

## 1 INTRODUCTION

Viroids, minimal known pathogens with autonomous replication, are naturally found infecting plants (Flores et al., 2015). The mature form is a covalently closed single‐stranded small RNA molecule (246–434 nucleotides [nt]) with a highly compact structure (Steger & Perreault, 2016). The vast majority of viroids replicate in the nucleus, have a central conserved region (CCR), and are classified in the family *Pospiviroidae*, while chloroplastic viroids, presenting self‐cleavage capabilities, comprise the family *Avsunviroidae* (Di Serio et al., 2014). *Hop stunt viroid* (HSVd) is a nuclear replicating viroid that has been reported in a wide range of hosts. The disentangling of the molecular biology and physical properties of viroids has been mainly carried out using *Potato spindle tuber viroid*, the first viroid discovered and type species of the family *Pospiviroidae* (Owens, 2007). However, the characterization of other members of the family has provided additional information about how these RNA pathogens interact with the host machinery (Adkar‐Purushothama & Perreault, 2019a) and has contributed to a large number of innovative techniques in RNA biology research (Steger & Riesner, 2018). In particular, multiple studies using HSVd as a model have shed light on different aspects of viroid–host interactions mainly related to long‐distance movement, resistance to RNA silencing, pathogenesis, and epigenetic alterations. As the symptomatology, economic significance, and phylogenetic relationships have been extensively reviewed (Hataya et al., 2017), we mainly focus on less‐covered aspects that occur during HSVd–host interaction such as the pathogenic and epigenetic processes.

## 2 DISCOVERY, HOSTS, AND SYMPTOMATOLOGY

**FIGURE 1:**
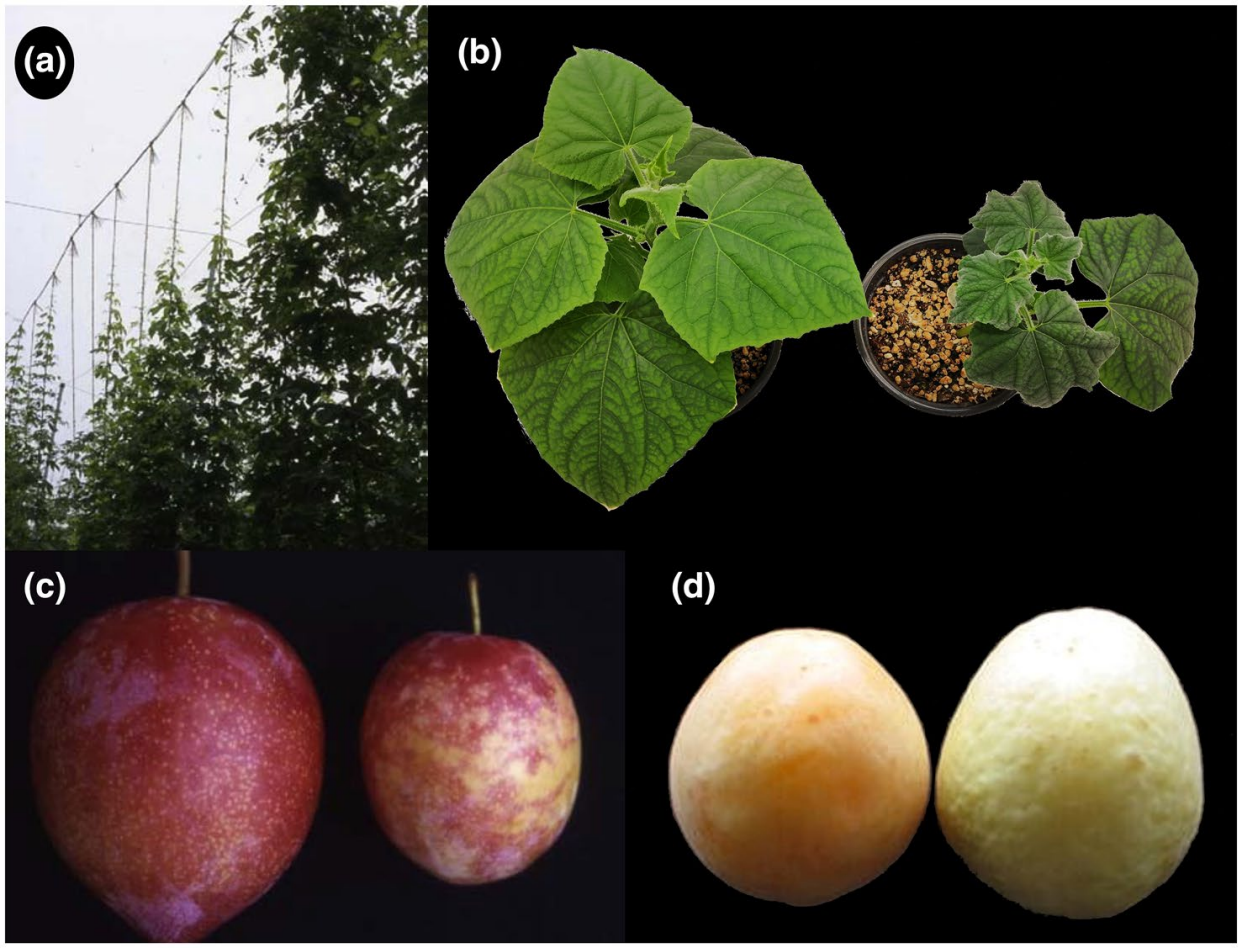
Symptomatology associated with HSVd infection. (a) Hop plants in field conditions showing abnormal dwarfing of bines. (b) Cucumber plant mock‐inoculated (left) or inoculated with HSVd (right), the last one showing clear stunting and leaf distortion. (c) Dapple fruit disease in plum cv. Shiho. Plum fruit with irregular reddish blotches on the pericarp caused by HSVd (right) compared with healthy (left) plum. (d) Apricot disease caused by HSVd (right) and healthy apricot (left). Images (a) and (c) are reproduced from Hataya et al. (2017) with permission of Elsevier Books and (d) from Amari et al. (2007), with permission of Springer Nature

*Hop stunt viroid* was first identified in hop (*Humulus lupulus*) plants showing abnormal dwarfing of bines (Sasaki & Shikata, 1977). This hop disease was initially described in Japan in the 1940s–1950s and it was estimated that between 10% and 20% of hop gardens were infected in the 1970s, causing important economic losses (Hataya et al., 2017). Since the late 1980s, hop stunt disease has been sporadic in Japan, but new epidemics have been reported in China (Guo et al., 2008), the United States (Eastwell & Nelson, 2007; Kappagantu et al., 2017a), and Slovenia (Radisek et al., 2012). Typical stunting symptoms in hops are only evident after years of being infected with HSVd (Figure 1a). Therefore, the discovery that cucumber could be an indicator host of this disease, developing symptoms in the range of weeks (Figure 1b), was key to characterize the properties of HSVd as the causal agent (Sasaki & Shikata, 1977). A cucumber disease with similar symptomatology was reported in the Netherlands, and the causal agent was also found to be a viroid, which was named *Cucumber pale fruit viroid* (CPFVd) (van Dorst & Peters, 1974). The sequencing of HSVd (Ohno et al., 1983) resulted in a 297 nt circular RNA with a rod‐like structure, which was 95% homologous to CPFVd, and thus the latter was considered an HSVd isolate and not a different viroid species (Sano et al., 1984). Based on phylogenetic analysis, a grapevine origin was proposed for the HSVd isolate causing hop stunt disease (Sano et al., 2001), which was later confirmed in a study covering 15 years of evolutionary analysis (Kawaguchi‐Ito et al., 2009).

HSVd has been identified in a wide range of host plants of the families Moraceae (fig and mulberry), Rosaceae (almond, apple, apricot, cherry, peach, pear, and plum), Anacardiaceae (pistachio) (Maddahian et al., 2019), Malvaceae (*Hibiscus rosa‐sinensis*) (Luigi et al., 2013), and Rutaceae (*Citrus* spp.) (Hataya et al., 2017; Vamenani et al., 2019). HSVd infection in most of these hosts is symptomless but other diseases have been identified in fruit trees associated with diverse isolates of this viroid (Di Serio et al., 2018). In particular, cachexia in citrus (Semancik et al., 1988) and fruit diseases in trees of the genus *Prunus*: plum and peach (Sano et al., 1989) (Figure 1c), apricot (Amari et al., 2007) (Figure 1d), and sweet cherry (Xu et al., 2017).

## 3 SURVEY AND DIAGNOSTIC METHODS

Initially, the presence of HSVd was detected by bioassays in the sensitive cucumber cv. Suyo (Sasaki & Shikata, 1977). Nucleic acids or sap extracts were mechanically inoculated in this indicator variety of cucumber and severe stunting symptoms appeared after 3–4 weeks when plants were grown at 25–30 °C (Figure 1b). The subsequent development of analytical techniques based on polyacrylamide gel electrophoresis (PAGE) allowed the characterization of the infectious agent. This enabled a faster identification of infected plants without waiting for symptom expression in an indicator host (see Hanold & Vadamalai, 2017, for review). However, even though two‐dimensional PAGE and sequential PAGE could confirm the presence of a viroid‐like agent, further sequencing is required to identify HSVd, making it extremely laborious to detect the pathogen unequivocally. That problem was solved with the development of hybridization‐based methods (Li et al., 1995; Owens & Diener, 1981; Pallas et al., 2017). The use of HSVd‐specific probes was crucial for the diagnosis of this pathogen, especially in fruit trees (Astruc et al., 1996). Dot blot and tissue printing hybridization with nonisotopic probes have been used for analysing large numbers of samples and therefore have been very useful for tracking HSVd infection in fruit trees in field conditions (Amari et al., 2001a; Astruc et al., 1996; Cañizares et al., 1998, 1999; Hassan et al., 2009; Mandic et al., 2008). Despite being economical and reliable in optimized conditions, hybridization methods have one important drawback: a limited sensitivity. A more sensitive method applied to HSVd detection has been reverse transcription (RT)‐PCR (Luigi & Faggioli, 2013; Yang et al., 1992) and a real‐time variant has been established for differentiating between HSVd variants in infected samples (Loconsole et al., 2013). Additionally, a rapid isothermal assay has been set up for an easy and sensitive survey of HSVd disease (Kappagantu et al., 2017b). Recently, high‐throughput sequencing technologies have enabled the detection of HSVd from small RNA deep sequencing (Su et al., 2015) and transcriptomic data (Jo et al., 2017). The use of these techniques could contribute to the discovery of HSVd in new hosts and potentially reveal new sequence variants.

## 4 GENOMIC VARIATION

The genome of members of the *Pospiviroidae* family is divided into five structural domains (Keese & Symons, 1985): two terminal regions, left (TL) and right (TR), pathogenic (P), variable (V), and a central domain (C) that contains a CCR, which is the characteristic hallmark of all members of the family (Di Serio et al., 2014). Additionally, HSVd contains a terminal conserved hairpin (TCH) that is also present in members of the genus *Cocadviroid* and the type species of the genus *Coleviroid* (*Coleus blumei viroid 1*) (Di Serio et al., 2017). The widespread distribution of HSVd in many hosts and its sequence diversity is reflected in a large number of HSVd sequences deposited in public repositories (382 nonredundant variants from at least 17 species) (Jo et al., 2017). The mutation rate of HSVd must be similar to that of other nuclear‐replicating viroids and RNA viruses (López‐Carrasco et al., 2017), but not as high as that of viroids of the family *Avsunvioridae* (Gago et al., 2009). Factors determining the mutation rate of nuclear viroids are mainly controlled by the host (López‐Carrasco et al., 2017). The analysis of sequence variation in different hosts has found a certain bias that suggests the adaptation of viroids to a host over a prolonged time (Kawaguchi‐Ito et al., 2009). Thus, the diversity of HSVd isolates may be explained by a low‐fidelity replication of RNA polymerase II, which is forced to accept viroid RNAs as template, in combination with its host range. Several phylogenetic analyses have grouped HSVd sequences into five different groups (Amari et al., 2001b; Jo et al., 2017; Kofalvi et al., 1997). Three of these groups (plum‐type, hop‐type, and citrus‐type) contain sequence variants predominantly from one host or related species. In contrast, the other two groups present signatures that suggest a recombinant origin (plum‐citrus‐type and plum‐hop/cit3‐type) (Kofalvi et al., 1997). The molecular characterization of Chinese HSVd isolates revealed a new phylogenetic group and a possible cross‐transmission between grapevine and stone fruits (Zhang et al., 2012). The size of HSVd has ranged from 295 to 303 nt in most of the sequence variants analysed so far. Yang et al. (2008) identified a unique variant with a tandem 15‐nucleotide repeat from a naturally infected plum tree. However, this sequence variant was not stably maintained in cucumber and thus its biological significance remains to be proved.

## 5 REPLICATION AND MOVEMENT

**FIGURE 2:**
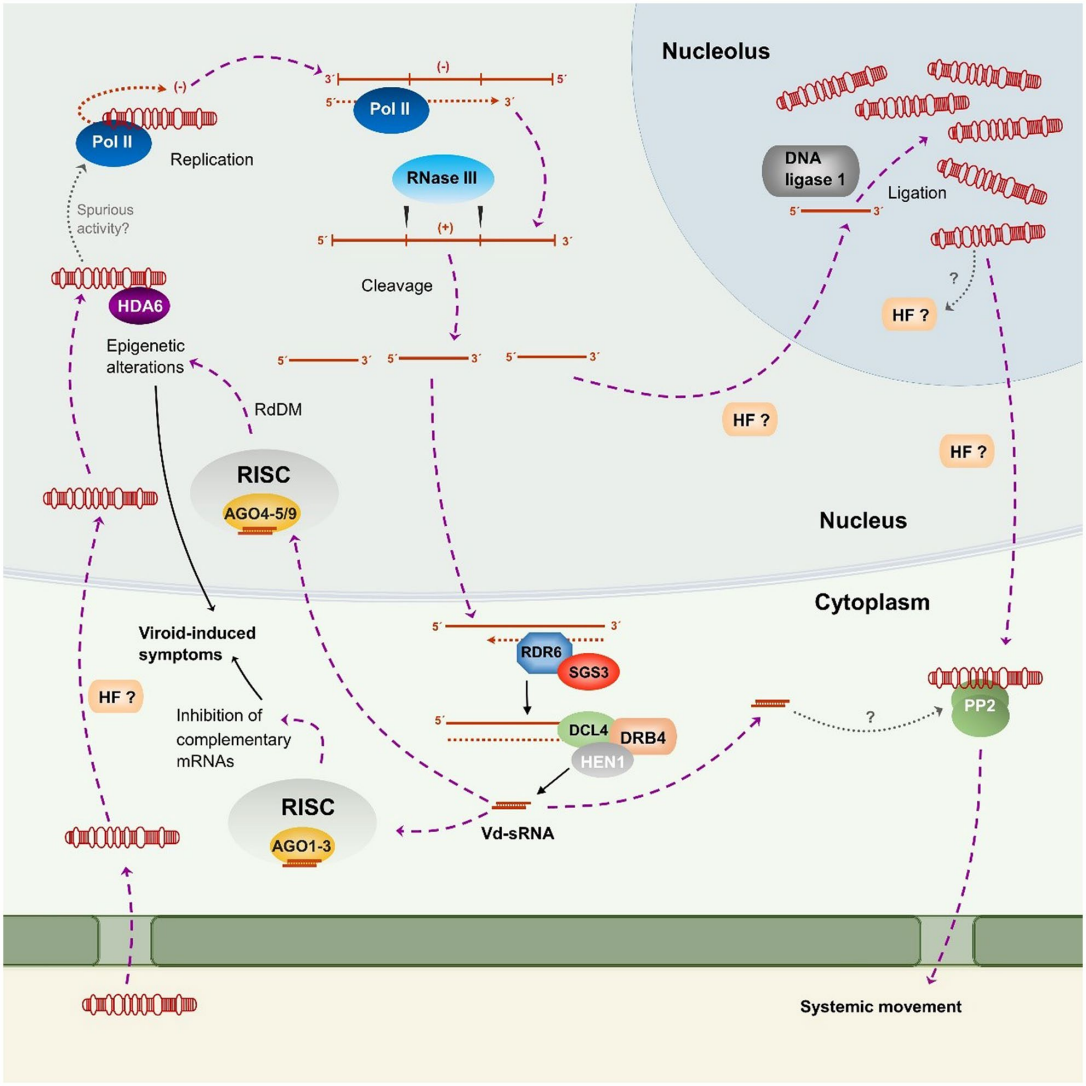
Proposed model of HSVd cell cycle and interactions with host factors. HSVd genomic RNA enters the cell through the plasmodesmata and it is trafficked to the nucleus, probably assisted by unidentified host factors. In the nucleus, HSVd subverts the activity of histone deacetylase 6 (HDA6) and causes epigenetic alterations. The subversion of HDA6 may favour a spurious recognition of the genomic HSVd RNA by the DNA‐dependent‐RNA polymerase II (Pol II). HSVd replication follows an asymmetric rolling circle in which Pol II transcribes HSVd RNAs. Oligomeric RNAs are cleaved by a host enzyme with RNase III activity and the resultant monomers are circularized by DNA ligase I, probably in the nucleolus, in which it is assumed that mature HSVd molecules accumulate. Other HSVd replication intermediates are recognized as aberrant transcripts and enter the *trans*‐acting small interfering RNA (tasiRNA) biogenesis pathway. Therefore, these RNAs are trafficked to the cytoplasm, transcribed by RDR6, and processed by DCL4 into viroid‐derived small RNAs (vd‐sRNAs) that are loaded into different AGO proteins. Vd‐sRNAs of 21–22 nucleotides are loaded in AGO 1‐3 in the RISC complex and direct the inhibition of host mRNA transcripts in the cytoplasm. Moreover, vd‐sRNAs of 24 nucleotides are primarily responsible for RNA‐directed DNA methylation (RdDM) in the nucleus. Thus, HSVd‐sRNAs cause transcriptional (nucleus) and posttranscriptional (cytoplasm) alterations that might account for the viroid‐induced symptoms. Mature HSVd genomic RNAs are exported to the phloem by forming a ribonucleoprotein complex with the phloem protein 2 (PP2). Additionally, the systemic movement of HSVd‐sRNA may also be possible due to the interaction with PP2

To replicate in host cells and eventually move to distal parts of the plant, viroids must interact with host factors and functionally subvert their activity (Ding, 2009). As with the rest of the members of the family *Pospiviroidae*, HSVd replicates in the nucleus through an asymmetric rolling circle pathway (Flores et al., 2004). In this pathway, the mature viroid is transcribed to linear oligomeric RNA intermediates that are used as a template for the transcription of RNAs of the same polarity as the initial molecule (+ polarity), subsequently cleaved and circularized into mature monomers (Figure 2). Early studies with longer‐than‐unit clones of HSVd (Meshi et al., 1985) revealed that the CCR may be involved in the processing of the oligomeric (+) strands through hairpin I, a structured motif that can be formed by the upper CCR strand and flanking nucleotides during thermal denaturation (Riesner et al., 1979). Evidence supporting which enzyme could be involved in the replication of nuclear‐viroids was first obtained with cucumber pale fruit viroid, a sequence variant of HSVd (Mühlbach & Sänger, 1979). It was shown that viroid replication in highly purified nuclei was specifically inhibited by α‐amanitin, a toxin specific to RNA polymerase II. Therefore, replication is not carried out by an RNA‐dependent polymerase; instead, host DNA‐dependent RNA polymerase II is subverted to accept viroid RNA as a template. Further experiments using other nuclear‐replicating viroids indicated that the cleavage of the oligomeric RNA intermediates would be carried out by a host type III RNase (Gas et al., 2007) and the resultant monomers are probably circularized by host DNA ligase 1 (Nohales et al., 2012). It is assumed that the (+)‐polarity strand of HSVd is accumulated in the nucleolus, as has been demonstrated in the type species PSTVd (Qi & Ding, 2003) and other members of the family (Bonfiglioli et al., 1996). Experimental evidence supports the existence of *cis*‐acting signals embedded in the viroid sequence that are required for nuclear targeting (Gómez & Pallás, 2012; Zhao et al., 2001). However, it remains unclear how exactly the (+)‐polarity strand could be selectively trafficked to the nucleolus. Curiously, a nucleolar localization signal was identified in a peptide derived from the HSVd sequence (Gómez & Pallás, 2007a). However, neither in vivo detection of this peptide nor experimental evidence about its functional relevance has been reported yet.

The ability of HSVd to infect cucurbitaceous hosts has been key for studying its systemic movement as it is relatively easy to obtain phloem exudates from these plants. Because of that, HSVd has been categorized as the viroid of choice for biochemical studies of long‐distance viroid transport (Owens, 2007). At the beginning of this century, it was already known that viroids used cellular mechanisms for systemic trafficking (Palukaitis, 1987; Zhu et al., 2002), although it was not known whether viroids move systemically as free RNA or form ribonucleoprotein complexes and, in general, the plant mechanisms involved in long distance RNA movement remained unclear (Ueki & Citovsky, 2001). However, in 2001, two independent research groups reported interaction in vitro between HSVd and the phloem protein 2 (PP2), which is a dimeric lectin and the most abundant component of the cucumber phloem exudate (Gómez & Pallás, 2001; Owens et al., 2001). The formation of the ribonucleoprotein complex in vivo was further confirmed by immunoprecipitation using a polyclonal serum against PP2. Moreover, the movement of this HSVd–PP2 complex was studied using intergeneric graft assays of a pumpkin scion, a plant that is not a host of HSVd, in an infected cucumber rootstock. Interestingly, the ribonucleotide complex was translocated and even symptoms of HSVd infection were observed in the scion despite being a nonhost plant (Gómez & Pallás, 2004). Additionally, a double‐stranded or highly structured RNA‐binding motif was identified in the PP2 sequence and it was reported that this lectin is the only protein in the phloem of cucumber able to bind HSVd. However, in melon, HSVd was additionally bound to other phloem lectins of 17 and 14 kDa, which suggested that different phloem proteins might be involved in viroid spread depending on the host (Gómez et al., 2004). Other phloem proteins have been suggested to be involved in the systemic transport of these pathogenic RNAs (Solovyev et al., 2013). Remarkably, several phloem proteins have been proven to have RNA‐binding capabilities to facilitate RNA trafficking through the phloem (Ham & Lucas, 2017; Pallas & Gómez, 2013). One of those factors has been proposed to be necessary to overcome the phloem restriction of viroids in three citrus hosts (Bani‐Hashemian et al., 2015).

*Nicotiana benthamiana* has been employed routinely as an experimental model in the study of plant–pathogen interactions. However, infectivity of HSVd in this host, although possible, appeared less efficient than in other ones. Inoculation attempts with nucleic acid preparations from viroid‐infected plants or HSVd dimeric transcripts failed to produce an efficient and reproducible systemic infection. To determine if this situation could be due to a defect in replication or systemic movement, transgenic plants of *N. benthamiana* expressing dimeric HSVd (+) RNAs (HSVd‐Nb plants) were obtained (Gómez & Pallás, 2006). HSVd was correctly processed into circular forms and grafting assays showed that movement to distal parts was possible. Therefore, deficiencies in the interaction with other host factors were proposed to explain the deficient infectivity rate observed in *N. benthamiana* plants. The most studied model plant is *Arabidopsis thaliana*, but systemic infection has not been possible with any viroid, and failed even when using the highly efficient *Agrobacterium*‐mediated inoculation method (Daròs & Flores, 2004). To gain insights into the limiting factors of viroid infection, Daròs and Flores (2004) obtained transgenic *Arabidopsis* expressing the dimeric (+) strand transcripts of the type member of each genus of the family *Pospiviroidae*. Northern blot hybridization revealed the correct processing to the circular monomer in all cases, and multimeric (−) RNAs of HSVd were also detected, indicating that the first RNA–RNA transcription of the rolling‐circle mechanism occurs in *Arabidopsis*. Furthermore, the second step of the rolling‐circle is possible because transgenic *Arabidopsis* expressing HSVd dimeric (−) transcripts accumulated the circular (+) monomers. These results suggest that the inability of HSVd to infect *Arabidopsis* may be related to systemic movement, although it cannot be excluded that low levels of replication might be hindering viroid spread.

## 6 PATHOGENESIS

How viroids induce disease has been a prevalent question in the field. Early studies suggested a sequence‐specificity of the symptoms, as point changes could greatly influence the degree of severity (Dickson et al., 1979). In the case of HSVd, sequence variants causing mild and severe symptoms have been identified (Xia et al., 2017). For example, a motif of five or six nucleotides found in the variable domain was associated with cachexia symptoms in citrus (Reanwarakorn & Semancik, 1998, 1999). Site‐directed mutagenesis of this cachexia motif could transform a severe strain into mild or asymptomatic, therefore demonstrating that it effectively modulates the symptom severity in citrus and may even suppress symptom expression (Serra et al., 2008). Additionally, transcriptomic changes induced by HSVd infection have been studied in cucumber (Xia et al., 2017) and hop (Kappagantu et al., 2017a). Results have shown a complex array of alterations, but an up‐regulation of basal defence genes, as well as the down‐regulation of genes related to essential metabolic processes, were described in both cases.

In recent decades, the demonstration that viroids trigger RNA silencing mechanisms in infected cells has supported an emerging notion linking viroid‐induced RNA silencing and pathogenesis (Gómez et al., 2009; Rodio et al., 2007; Sano et al., 2010; Wang et al., 2004). This attractive pathogenicity model proposes that viroid‐derived small RNAs (vd‐sRNAs) can mediate the specific degradation of certain endogenous transcripts, promoting the phenotypic alterations recognized as symptoms (Wang et al., 2004). Since the demonstration that viroid RNAs were substrates for Dicer‐like enzymes and cleaved into 20–24‐nt vd‐sRNA (Papaefthimiou et al., 2001), a large body of evidence has revealed that these small pathogenic RNAs are inducers and targets of the RNA silencing machinery (see Adkar‐Purushothama & Perreault, 2019a, for a review). Studies using HSVd as a model have been crucial to establish the dependence of RNA silencing mechanisms for viroid‐induced symptoms (Gómez et al., 2008; Zhang et al., 2020) and have shed light on the pathways involved in vd‐sRNA biogenesis (Gómez et al., 2009) and the resistance of mature forms to RISC‐mediated cleavage (Gómez & Pallás, 2007b).

First, transgenic HSVd‐Nb plants were used to propose that mature HSVd forms are resistant to RNA silencing (Gómez & Pallás, 2007b). A reporter construct, which consisted of a full‐length HSVd RNA fused to green fluorescent protein (GFP)‐mRNA (HSVd‐GFP), was agroinfiltrated in HSVd‐Nb plants, and its expression was silenced as a consequence of the vd‐sRNA activity (able to interact with the RNA silencing complexes and mediate the degradation of the linear HSVd‐RNA fused to the GFP transcripts). However, in these restrictive conditions, circular HSVd molecules were able to traffic through intergeneric grafts, indicating that the mature forms of HSVd possess certain resistance to RNA silencing‐mediated degradation. Similar results were obtained using PSTVd as a model (Itaya et al., 2007). Therefore, it was established that the highly packed structure of viroids is not only important for the interaction with host factors but also necessary to escape from RNA silencing, constituting a major constraint to viroid evolution (Catalán et al., 2019; Elena et al., 2009). HSVd was also employed as a valuable experimental model to analyse the biogenesis of vd‐sRNAs. High‐throughput sequencing of small RNAs in HSVd‐infected grapevine (Navarro et al., 2009) and cucumber (Martinez et al., 2010) indicated that the totality of the HSVd RNA genome contributes to the formation of vd‐sRNAs, predominantly of 21, 22, and 24 nt, but the accumulation profile showed that these small RNAs aligned preferentially in specific regions or hot spots in the viroid genome. Moreover, HSVd‐sRNAs derived almost equally from the (+) and (−) genomic strands (Castellano et al., 2016a; Martinez et al., 2010; Navarro et al., 2009; Su et al., 2015; Zhang et al., 2020). This fact suggested that HSVd sRNAs must be mainly originated from double‐stranded RNA viroid replication intermediates (Gómez et al., 2009; Martinez et al., 2010). Remarkably, phloem vd‐sRNAs accumulated preferentially as 22‐nt species with a consensus sequence over‐represented. This bias in size and sequence in the HSVd vd‐sRNA population recovered from phloem exudate suggests the existence of a selective trafficking of vd‐sRNAs to the phloem tissue of infected cucumber plants.

Regarding the interplay between HSVd pathogenesis and RNA silencing, it was initially observed that symptom expression in *N. benthamiana* plants is dependent on RNA‐dependent RNA polymerase 6 (RDR6) activity (Gomez et al., 2008). In that work, graft‐infected plants in which *RDR6* (a key component of the RNA silencing machinery) was constitutively silenced (*rdr6i‐Nb*) were unable to develop symptoms even though the HSVd mature forms accumulated at levels comparable to those observed in infected controls with symptoms. These results were reinforced by the observation that HSVd‐Nb plants showing severe symptoms at 28 °C were symptomless when maintained at low temperatures (14 °C) at which the RNA silencing pathways are inhibited (Gomez et al., 2008). Interestingly, hybridization assays revealed that HSVd circular and linear monomeric forms accumulated at similar levels in HSVd‐Nb plants at both tested temperatures. Thus, it was concluded that symptom expression is dependent on an active state of the viroid‐specific RNA silencing pathways and independent of mature HSVd accumulation levels. However, studies with PSTVd have found a higher viroid accumulation at early stages when *RDR6* is silenced in *N. benthamiana* (Adkar‐Purushothama & Perreault, 2019b; Di Serio et al., 2010) and lower in tomato (Naoi et al., 2020), with no differences in the symptomatology. Based on these studies, a defensive role in protecting the shoot apical meristem against PSTVd invasion has been attributed to RDR6, but it is unclear if it has this same function in the case of HSVd. It could be hypothesized that despite being of the same family, HSVd and PSTVd may interact differently with the silencing machinery, and this could explain why the suppression of *RDR6* has contrasting effects in both cases. Further studies are required to determine the whole picture.

Finally, and based on multiple similarities between viroid replication and *trans*‐acting small interfering RNA tasiRNA processing, it was proposed that the pathogenesis process associated with HSVd infection might be related to the accumulation of vd‐sRNAs generated as result from the incorporation of viroid replication intermediates in the tasiRNA biogenesis pathway (Gomez et al., 2009).

## 7 EPIGENETIC ALTERATIONS

Evidence that viroids can affect the methylation status of the host genome was pioneering and directed the discovery of the RNA‐directed DNA methylation (RdDM) mechanism (Wassenegger et al., 1994). However, later experimental insights gathered while working with HSVd revealed that viroids could additionally trigger epigenetic alterations in a noncanonical RdDM‐dependent manner. In cucumber plants a high accumulation of ribosomal RNA (rRNA) precursors was observed on HSVd infection. This phenomenon was correlated with a decrease in DNA methylation in the promoter region of the rRNA genes (Martinez et al., 2014). A similar pattern of loss of cytosine methylation (5mC) of usually silenced rRNA genes was detected in HSVd‐Nb plants, indicating that it might be a generalized phenomenon in HSVd pathogenesis (Castellano et al., 2015).

Nonetheless, a mechanistic explication for this phenomenon could not be outlined until the in vitro and in vivo interaction of HSVd with histone‐deacetylase 6 (HDA6) was reported, which is responsible for the removal of acetyl groups from the N‐terminal part of the core histones (H2A, H2B, H3, and H4) and gives a tag for epigenetic repression (Liu et al., 2012a, 2012b). HDA6 has been associated with the transcriptional repression of specific targets, such as rDNA repeats or complex transgenes (Liu et al., 2012a). Moreover, the transient silencing of cucumber HDA6 favoured HSVd accumulation while transient overexpression of recombinant HDA6 reverted the hypomethylation status of rDNA, characteristic of HSVd‐infected plants, and reduced viroid accumulation (Castellano et al., 2016b). Those observations led to the proposal that HSVd recruits and functionally subverts HDA6 to promote the host epigenetic changes in rRNA genes produced during viroid pathogenesis. Interestingly, the lack of HDA6 activity has been associated with spurious RNA polymerase II transcription of nonconventional rDNA templates (usually transcribed by RNA polymerase I) (Earley et al., 2010). In HSVd‐infected plants, it was reported an overaccumulation of pre‐rRNAs and small RNAs derived from ribosomal transcripts indicating an unusual transcriptional environment (Castellano et al., 2015, 2016b; Martinez et al., 2014). Therefore, it was proposed that the HDA6‐recruitment mediated by HSVd may promote spurious RNA polymerase II activity that could favour the transcription of noncanonical templates, thereby improving viroid replication.

In addition to these alterations in vegetative tissues, HSVd infection is also associated with drastic changes in gametophyte development (Castellano et al., 2016a). It was observed that in pollen grains the accumulation of HSVd RNA induces a decondensation of the generative nucleus that correlates with a dynamic demethylation of repetitive regions in the host genome (Castellano et al., 2016a). Therefore, the authors proposed that HSVd infection impairs the epigenetic control of these repetitive regions, primarily rRNA genes and transposable elements, in gametic cells of cucumber. That represents a previously undescribed phenomenon resulting from pathogen infection in this reproductive tissue.

## 8 CONCLUSION AND FURTHER PERSPECTIVES

The large number of studies involving HSVd have resulted in a considerable knowledge about the replication, systemic movement, and host range of this ubiquitous pathogen, as well as concerning its interaction with RNA silencing and epigenetic mechanisms of the host. HSVd might be the second most studied viroid after PSTVd. However, some aspects of HSVd pathogenesis are yet to be unravelled. For example, host transcripts effectively silenced by HSVd‐sRNAs have not been described yet despite the well‐proved relation of viroid pathogenesis with RNA silencing (Gómez et al., 2009; Rodio et al., 2007; Sano et al., 2010; Wang et al., 2004). Moreover, the genome‐wide alterations of 5mC levels in the genome of HSVd‐infected plants are unknown. It could be speculated that the cytosine methylation in other regions targeted by HDA6, such as repetitive DNA encoding transposable elements (Liu et al., 2012b), might also be affected, as it was suggested from the deep‐sequencing data of small RNAs in the cucumber male gametophyte (Castellano et al., 2016a). For that reason, further studies, like whole‐genome bisulphite methylation, would be required to characterize the extent of the modifications that may be triggered by the subversion of HDA6 by HSVd (Castellano et al., 2016b). Additionally, the presence of viroid‐induced modifications in cucumber pollen raises questions about the possible heritability of these alterations (Castellano et al., 2016a). On the other hand, a more global study (at temporal scale during infection) focused on the viroid‐induced alterations in the transcriptional landscape of the host could provide valuable information about the endogenous regulatory pathways subverted and/or redirected by HSVd during the infectious process. Finally, the analysis of sequence diversity and site‐directed mutagenesis in infectious cDNA clones of HSVd (Marquez‐Molins et al., 2019) could provide more insights into the sequence–function relations of viroids. For instance, sequence motifs involved in movement, or in general with the interaction with host factors, might be discovered using this approach. Interestingly, an open question is whether interactions with host mechanisms (like RNA silencing or those that maintain the epigenetic stability of the genome) provide an adaptive advantage for viroids or, conversely, viroid‐induced pathogenesis is only a side effect with no implications in viroid fitness.

## CONFLICT OF INTEREST

The authors declare no conflict of interest.

## Data Availability

Data sharing is not applicable to this article as no new data were created or analysed in this study.

## References

[mpp13022-bib-0001] Adkar‐Purushothama, C.R. & Perreault, J.‐P. (2019a) Current overview on viroid–host interactions. WIREs RNA, 11, e1570. 31642206 10.1002/wrna.1570

[mpp13022-bib-0002] Adkar‐Purushothama, C.R. & Perreault, J.P. (2019b) Suppression of RNA‐dependent RNA polymerase 6 favors the accumulation of potato spindle tuber viroid in nicotiana benthamiana. Viruses, 11, 345. 10.3390/v11040345 PMC6520914 31013994

[mpp13022-bib-0003] Amari, K. , Cañizares, M.C. , Pallás, V. , Myrta, A. , Sabanadzovic, S. & Terlizzi, B.D. (2001a) Tracking hop stunt viroid infection in apricot trees during a whole year by non‐isotopic tissue printing hybridisation. Acta Horticulturae, 550, 315–319.

[mpp13022-bib-0004] Amari, K. , Gomez, G. , Myrta, A. , Di Terlizzi, B. & Pallás, V. (2001b) The molecular characterization of 16 new sequence variants of hop stunt viroid reveals the existence of invariable regions and a conserved hammerhead‐like structure on the viroid molecule. Journal of General Virology, 82, 953–962. 10.1099/0022-1317-82-4-953 11257203

[mpp13022-bib-0005] Amari, K. , Ruiz, D. , Gómez, G. , Sánchez‐Pina, M.A. , Pallás, V. & Egea, J. (2007) An important new apricot disease in Spain is associated with hop stunt viroid infection. European Journal of Plant Pathology, 118, 173–181.

[mpp13022-bib-0006] Astruc, N. , Marcos, J.F. , Macquaire, G. , Candresse, T. & Pallás, V. (1996) Studies on the diagnosis of hop stunt viroid in fruit trees: Identification of new hosts and application of a nucleic acid extraction procedure based on non‐organic solvents. European Journal of Plant Pathology, 102, 837–846.

[mpp13022-bib-0007] Bani‐Hashemian, S.M. , Pensabene‐Bellavia, G. , Duran‐Vila, N. & Serra, P. (2015) Phloem restriction of viroids in three citrus hosts is overcome by grafting with Etrog citron: Potential involvement of a translocatable factor. Journal of General Virology, 96, 2405–2410. 10.1099/vir.0.000154 25888624

[mpp13022-bib-0008] Bonfiglioli, R.G. , Webb, D.R. & Symons, R.H. (1996) Tissue and intra‐cellular distribution of coconut cadang cadang viroid and citrus exocortis viroid determined by in situ hybridization and confocal laser scanning and transmission electron microscopy. The Plant Journal, 9, 457–465.

[mpp13022-bib-0009] Cañizares, M.C. , Marcos, J.F. & Pallás, V. (1998) Studies on the incidence of hop stunt viroid in apricot trees (*Prunus armeniaca*) by using an easy and short extraction method to analyze a large number of samples. Acta Horticulturae, 472, 581–585.

[mpp13022-bib-0010] Cañizares, M.C. , Marcos, J.F. & Pallás, V. (1999) Molecular characterization of an almond isolate of hop stunt viroid (HSVd) and conditions for eliminating spurious hybridization in its diagnosis in almond samples. European Journal of Plant Pathology, 105, 553–558.

[mpp13022-bib-0011] Castellano, M. , Martinez, G. , Pallás, V. & Gómez, G. (2015) Alterations in host DNA methylation in response to constitutive expression of hop stunt viroid RNA in *Nicotiana benthamiana* plants. Plant Pathology, 64, 1247–1257.

[mpp13022-bib-0012] Castellano, M. , Martinez, G. , Marques, M.C. , Moreno‐Romero, J. , Köhler, C. , Pallas, V. et al. (2016a) Changes in the DNA methylation pattern of the host male gametophyte of viroid‐infected cucumber plants. Journal of Experimental Botany, 67, 5857–5868. 27697787 10.1093/jxb/erw353 PMC5066502

[mpp13022-bib-0013] Castellano, M. , Pallas, V. & Gomez, G. (2016b) A pathogenic long noncoding RNA redesigns the epigenetic landscape of the infected cells by subverting host histone deacetylase 6 activity. New Phytologist, 211, 1311–1322. 10.1111/nph.14001 27174164

[mpp13022-bib-0014] Catalán, P. , Elena, S.F. , Cuesta, J.A. & Manrubia, S. (2019) Parsimonious scenario for the emergence of viroid‐like replicons de novo. Viruses, 11, 425. 10.3390/v11050425 PMC6563258 31075860

[mpp13022-bib-0015] Daròs, J.‐A. & Flores, R. (2004) *Arabidopsis thaliana* has the enzymatic machinery for replicating representative viroid species of the family *Pospiviroidae* . Proceedings of the National Academy of Sciences of the United States of America, 101, 6792–6797. 15096616 10.1073/pnas.0401090101 PMC404124

[mpp13022-bib-0016] Di Serio, F. , Ambrós, S. , Sano, T. , Flores, R. & Navarro, B. (2018) Viroid diseases in pome and stone fruit trees and Koch’s postulates: A critical assessment. Viruses, 10, 612. 10.3390/v10110612 PMC6265958 30405008

[mpp13022-bib-0017] Di Serio, F. , Flores, R. , Verhoeven, J.T.J. , Li, S.‐F. , Pallás, V. , Randles, J.W. et al. (2014) Current status of viroid taxonomy. Archives of Virology, 159, 3467–3478. 25216773 10.1007/s00705-014-2200-6

[mpp13022-bib-0018] Di Serio, F. , Martínez de Alba, A.‐E. , Navarro, B. , Gisel, A. & Flores, R. (2010) RNA‐dependent RNA polymerase 6 delays accumulation and precludes meristem invasion of a viroid that replicates in the nucleus. Journal of Virology, 84, 2477–2489. 20015979 10.1128/JVI.02336-09 PMC2820905

[mpp13022-bib-0019] Dickson, E. , Robertson, H.D. , Niblett, C.L. , Horst, R.K. & Zaitlin, M. (1979) Minor differences between nucleotide sequences of mild and severe strains of potato spindle tuber viroid. Nature, 277, 60–62.

[mpp13022-bib-0020] Ding, B. (2009) The biology of viroid–host interactions. Annual Review of Phytopathology, 47, 105–131. 10.1146/annurev-phyto-080508-081927 19400635

[mpp13022-bib-0104] Di Serio, F. , Navarro, B. & Flores, R. (2017) Origin and evolution of viroids In: Hadidi A., Flores R., Randles J. and Palukaitis P. (Eds.), Viroids and Satellites. Cambridge: Academic Press – Elsevier, pp. 125–134.

[mpp13022-bib-0093] van Dorst, H.J.M. & Peters, D. (1974) Some biological observations on pale fruit, a viroid‐incited disease of cucumber. Netherlands Journal of Plant Pathology, 80, 85–96.

[mpp13022-bib-0021] Earley, K.W. , Pontvianne, F. , Wierzbicki, A.T. , Blevins, T. , Tucker, S. , Costa‐Nunes, P. et al. (2010) Mechanisms of HDA6‐mediated rRNA gene silencing: Suppression of intergenic Pol II transcription and differential effects on maintenance versus siRNA‐directed cytosine methylation. Genes and Development, 24, 1119–1132. 20516197 10.1101/gad.1914110 PMC2878650

[mpp13022-bib-0022] Eastwell, K.C. & Nelson, M.E. (2007) Occurrence of viroids in commercial hop (*Humulus lupulus* L.) production areas of Washington State. Plant Health Progress, 8, 10.1094/PHP-2007-1127-01-RS

[mpp13022-bib-0023] Elena, S.F. , Gómez, G. & Daròs, J.A. (2009) Evolutionary constraints to viroid evolution. Viruses, 1, 241–254. 21994548 10.3390/v1020241 PMC3185485

[mpp13022-bib-0024] Flores, R. , Delgado, S. , Gas, M.E. , Carbonell, A. , Molina, D. , Gago, S. et al. (2004) Viroids: The minimal non‐coding RNAs with autonomous replication. FEBS Letters, 567, 42–48. 15165891 10.1016/j.febslet.2004.03.118

[mpp13022-bib-0025] Flores, R. , Minoia, S. , Carbonell, A. , Gisel, A. , Delgado, S. , López‐Carrasco, A. et al. (2015) Viroids, the simplest RNA replicons: How they manipulate their hosts for being propagated and how their hosts react for containing the infection. Virus Researchs, 209, 136–145. 10.1016/j.virusres.2015.02.027 25738582

[mpp13022-bib-0026] Gago, S. , Elena, S.F. , Flores, R. & Sanjuán, R. (2009) Extremely high mutation rate of a hammerhead viroid. Science, 323, 1308. 19265013 10.1126/science.1169202

[mpp13022-bib-0027] Gas, M.‐E. , Hernández, C. , Flores, R. & Daròs, J.‐A. (2007) Processing of nuclear viroids in vivo: An interplay between RNA conformations. PLoS Pathogens, 3, e182. 18052530 10.1371/journal.ppat.0030182 PMC2098832

[mpp13022-bib-0028] Gómez, G. , Martínez, G. & Pallás, V. (2008) Viroid‐induced symptoms in *Nicotiana benthamiana* plants are dependent on RDR6 activity. Plant Physiology, 148, 414–423. 18599649 10.1104/pp.108.120808 PMC2528107

[mpp13022-bib-0029] Gómez, G. , Martínez, G. & Pallás, V. (2009) Interplay between viroid‐induced pathogenesis and RNA silencing pathways. Trends in Plant Science, 14, 264–269. 19375972 10.1016/j.tplants.2009.03.002

[mpp13022-bib-0030] Gómez, G. & Pallás, V. (2001) Identification of an in vitro ribonucleoprotein complex between a viroid RNA and a phloem protein from cucumber plants. Molecular Plant‐Microbe Interactions, 14, 910–913. 11437265 10.1094/MPMI.2001.14.7.910

[mpp13022-bib-0031] Gómez, G. & Pallás, V. (2004) A long‐distance translocatable phloem protein from cucumber forms a ribonucleoprotein complex in vivo with hop stunt viroid RNA. Journal of Virology, 78, 10104–10110. 15331743 10.1128/JVI.78.18.10104-10110.2004 PMC514978

[mpp13022-bib-0032] Gómez, G. & Pallás, V. (2006) Hop stunt viroid is processed and translocated in transgenic *Nicotiana benthamiana* plants. Molecular Plant Pathology, 7, 511–517. 20507465 10.1111/j.1364-3703.2006.00356.x

[mpp13022-bib-0033] Gómez, G. & Pallás, V. (2007a) A peptide derived from a single‐modified viroid‐RNA can be used as an “in vivo” nucleolar marker. Journal of Virological Methods, 144, 169–171. 17570537 10.1016/j.jviromet.2007.04.009

[mpp13022-bib-0034] Gómez, G. & Pallás, V. (2007b) Mature monomeric forms of hop stunt viroid resist RNA silencing in transgenic plants. The Plant Journal, 51, 1041–1049. 17711417 10.1111/j.1365-313X.2007.03203.x

[mpp13022-bib-0035] Gómez, G. & Pallás, V. (2012) Studies on subcellular compartmentalization of plant pathogenic noncoding RNAs give new insights into the intracellular RNA‐traffic mechanisms. Plant Physiology, 159, 558–564. 22474218 10.1104/pp.112.195214 PMC3375924

[mpp13022-bib-0036] Gómez, G. , Torres, H. & Pallás, V. (2004) Identification of translocatable RNA‐binding phloem proteins from melon, potential components of the long‐distance RNA transport system. The Plant Journal, 41, 107–116. 10.1111/j.1365-313X.2004.02278.x 15610353

[mpp13022-bib-0037] Guo, L. , Liu, S. , Wu, Z. , Mu, L. , Xiang, B. & Li, S. (2008) Hop stunt viroid (HSVd) newly reported from hop in Xinjiang, China. Plant Pathology, 57, 764.

[mpp13022-bib-0038] Ham, B.‐K. & Lucas, W.J. (2017) Phloem‐mobile RNAs as systemic signaling sgents. Annual Review of Plant Biology, 68, 173–195. 10.1146/annurev-arplant-042916-041139 28125282

[mpp13022-bib-0039] Hanold, D. & Vadamalai, G. (2017) Gel electrophoresis In: Hadidi A., Randles J., Flores R. and Palukaitis P. (Eds.), Viroids and Satellites. Cambridge: Academic Press – Elsevier, pp. 357–367.

[mpp13022-bib-0040] Hassan, M. , Gomez, G. , Pallás, V. , Myrta, A. & Rysanek, P. (2009) Simultaneous detection and genetic variability of stone fruit viroids in the Czech Republic. European Journal of Plant Pathology, 124, 363–368.

[mpp13022-bib-0041] Hataya, T. , Tsushima, T. & Sano, T. (2017) Hop stunt viroid In: Hadidi A., Randles J., Flores R. and Palukaitis P. (Eds.), Viroids and Satellites. Cambridge: Academic Press ‐ Elsevier, pp. 199–210.

[mpp13022-bib-0042] Itaya, A. , Zhong, X. , Bundschuh, R. , Qi, Y. , Wang, Y. , Takeda, R. et al. (2007) A structured viroid RNA serves as a substrate for Dicer‐like cleavage to produce biologically active small RNAs but is resistant to RNA‐induced silencing complex‐mediated degradation. Journal of Virology, 81, 2980–2994. 17202210 10.1128/JVI.02339-06 PMC1865973

[mpp13022-bib-0043] Jo, Y. , Chu, H. , Kim, H. , Cho, J.K. , Lian, S. , Choi, H. et al. (2017) Comprehensive analysis of genomic variation of hop stunt viroid. European Journal of Plant Pathology, 148, 119–127.

[mpp13022-bib-0044] Kappagantu, M. , Bullock, J.M. , Nelson, M.E. & Eastwell, K.C. (2017a) Hop stunt viroid: Effect on host (*Humulus lupulus*) transcriptome and its interactions with hop powdery mildew (*Podospheara macularis*). Molecular Plant‐Microbe Interactions, 30, 842–851. 28703029 10.1094/MPMI-03-17-0071-R

[mpp13022-bib-0045] Kappagantu, M. , Villamor, D.E.V. , Bullock, J.M. & Eastwell, K.C. (2017b) A rapid isothermal assay for the detection of hop stunt viroid in hop plants (*Humulus lupulus*), and its application in disease surveys. Journal of Virological Methods, 245, 81–85. 28392409 10.1016/j.jviromet.2017.04.002

[mpp13022-bib-0046] Kawaguchi‐Ito, Y. , Li, S.F. , Tagawa, M. , Araki, H. , Goshono, M. , Yamamoto, S. et al. (2009) Cultivated grapevines represent a symptomless reservoir for the transmission of hop stunt viroid to hop crops: 15 years of evolutionary analysis. PLoS One, 4, e8386. 20041179 10.1371/journal.pone.0008386 PMC2793511

[mpp13022-bib-0047] Keese, P. & Symons, R.H. (1985) Domains in viroids: Evidence of intermolecular RNA rearrangements and their contribution to viroid evolution. Proceedings of the National Academy of Sciences of the United States of America, 82, 4582–4586. 3860809 10.1073/pnas.82.14.4582 PMC390429

[mpp13022-bib-0048] Kofalvi, S.A. , Marcos, J.F. , Cañizares, M.C. , Pallas, V. & Candresse, T. (1997) Hop stunt viroid (HSVd) sequence variants from *Prunus* species: evidence for recombination between HSVd isolates. Journal of General Virology, 78, 3177–3186. 10.1099/0022-1317-78-12-3177 9400968

[mpp13022-bib-0049] Li, S.‐F. , Onodera, S. , Sano, T. , Yoshida, K. , Wang, G.‐P. & Shikata, E. (1995) Gene diagnosis of viroids: comparisons of return‐PAGE and hybridization using DIG‐labeled DNA and RNA probes for practical diagnosis of hop stunt, citrus exocortis and apple scar skin viroids in their natural host plants. Japanese Journal of Phytopathology, 61, 381–390.

[mpp13022-bib-0050] Liu, X. , Luo, M. & Wu, K. (2012a) Epigenetic interplay of histone modifications and DNA methylation mediated by HDA6. Plant Signaling and Behavior, 7, 633–635. 22580702 10.4161/psb.19994 PMC3442857

[mpp13022-bib-0051] Liu, X. , Yu, C.W. , Duan, J. , Luo, M. , Wang, K. , Tian, G. et al. (2012b) HDA6 directly interacts with DNA methyltransferase MET1 and maintains transposable element silencing in *Arabidopsis* . Plant Physiology, 158, 119–129. 21994348 10.1104/pp.111.184275 PMC3252112

[mpp13022-bib-0052] Loconsole, G. , Önelge, N. , Yokomi, R.K. , Kubaa, R.A. , Savino, V. & Saponari, M. (2013) Rapid differentiation of citrus Hop stunt viroid variants by real‐time RT‐PCR and high resolution melting analysis. Molecular and Cellular Probes, 27, 221–229. 23932930 10.1016/j.mcp.2013.07.003

[mpp13022-bib-0053] López‐Carrasco, A. , Ballesteros, C. , Sentandreu, V. , Delgado, S. , Gago‐Zachert, S. , Flores, R. et al. (2017) Different rates of spontaneous mutation of chloroplastic and nuclear viroids as determined by high‐fidelity ultra‐deep sequencing. PLoS Pathogens, 13, e1006547. 28910391 10.1371/journal.ppat.1006547 PMC5614642

[mpp13022-bib-0054] Luigi, M. & Faggioli, F. (2013) Development of a quantitative real‐time RT‐PCR (qRT‐PCR) for the detection of hop stunt viroid. European Journal of Plant Pathology, 137, 231–235.

[mpp13022-bib-0055] Luigi, M. , Manglli, A. , Tomassoli, L. & Faggioli, F. (2013) First report of *Hop stunt viroid* in *Hibiscus rosa‐sinensis* in Italy. New Disease Reports, 27, 14.

[mpp13022-bib-0056] Maddahian, M. , Massumi, H. , Heydarnejad, J. , Hosseinipour, A. , Khezri, A. & Sano, T. (2019) Biological and molecular characterization of hop stunt viroid variants from pistachio trees in Iran. Journal of Phytopathology, 9, 1–11.

[mpp13022-bib-0057] Mandic, B. , Rwahnih, M.A. , Myrta, A. , Gomez, G. & Pallás, V. (2008) Incidence and genetic diversity of Peach latent mosaic viroid and Hop stunt viroid in stone fruits in Serbia. European Journal of Plant Pathology, 120, 167–176.

[mpp13022-bib-0058] Marquez‐Molins, J. , Navarro, J.A. , Pallas, V. & Gomez, G. (2019) Highly efficient construction of infectious viroid‐derived clones. Plant Methods, 15, 87. 31388344 10.1186/s13007-019-0470-4 PMC6670230

[mpp13022-bib-0059] Martinez, G. , Castellano, M. , Tortosa, M. , Pallas, V. & Gomez, G. (2014) A pathogenic non‐coding RNA induces changes in dynamic DNA methylation of ribosomal RNA genes in host plants. Nucleic Acids Research, 42, 1553–1562. 24178032 10.1093/nar/gkt968 PMC3919566

[mpp13022-bib-0060] Martinez, G. , Donaire, L. , Llave, C. , Pallas, V. & Gomez, G. (2010) High‐throughput sequencing of Hop stunt viroid‐derived small RNAs from cucumber leaves and phloem. Molecular Plant Pathology, 11, 347–359. 20447283 10.1111/j.1364-3703.2009.00608.x PMC6640512

[mpp13022-bib-0061] Meshi, T. , Ishikawa, M. , Watanabe, Y. , Yamaya, J. , Okada, Y. , Sano, T. et al. (1985) The sequence necessary for the infectivity of hop stunt viroid cDNA clones. Molecular and General Genetics, 200, 199–206.

[mpp13022-bib-0062] Mühlbach, H.P. & Sänger, H.L. (1979) Viroid replication is inhibited by α‐amanitin. Nature, 278, 185–188. 763366 10.1038/278185a0

[mpp13022-bib-0063] Naoi, T. , Kitabayashi, S. , Kasai, A. , Sugawara, K. , Adkar‐Purushothama, C.R. , Senda, M. et al. (2020) Suppression of RNA‐dependent RNA polymerase 6 in tomatoes allows potato spindle tuber viroid to invade basal part but not apical part including pluripotent stem cells of shoot apical meristem. PLoS One, 15, e0236481. 32716919 10.1371/journal.pone.0236481 PMC7384629

[mpp13022-bib-0064] Navarro, B. , Pantaleo, V. , Gisel, A. , Moxon, S. , Dalmary, T. , Bisztray, G. et al. (2009) Deep sequencing of viroid‐derived small RNAs from grapevine provides new insights on the role of RNA silencing in plant–viroid interaction. PLoS One, 4, e7686. 19890399 10.1371/journal.pone.0007686 PMC2767511

[mpp13022-bib-0065] Nohales, M.Á. , Flores, R. & Daròs, J.A. (2012) Viroid RNA redirects host DNA ligase 1 to act as an RNA ligase. Proceedings of the National Academy of Sciences of the United States of America, 109, 13805–13810. 22869737 10.1073/pnas.1206187109 PMC3427106

[mpp13022-bib-0066] Ohno, T. , Takamatsu, N. , Meshi, T. & Okada, Y. (1983) Hop stunt viroid: molecular cloning and nucleotide sequence of the complete cDNA copy. Nucleic Acids Research, 11, 6185–6197. 6312412 10.1093/nar/11.18.6185 PMC326366

[mpp13022-bib-0067] Owens, R.A. (2007) Potato spindle tuber viroid: the simplicity paradox resolved? Molecular Plant Pathology, 8, 549–560. 20507521 10.1111/j.1364-3703.2007.00418.x

[mpp13022-bib-0068] Owens, R.A. , Blackburn, M. & Ding, B. (2001) Possible involvement of the phloem lectin in long‐distance viroid movement. Molecular Plant‐Microbe Interactions, 14, 905–909. 11437264 10.1094/MPMI.2001.14.7.905

[mpp13022-bib-0069] Owens, R.A. & Diener, T.O. (1981) Sensitive and rapid diagnosis of *Potato spindle tuber viroid* disease by nucleic acid hybridization. Science, 213, 670–672. 17847478 10.1126/science.213.4508.670

[mpp13022-bib-0070] Pallas, V. & Gómez, G. (2013) Phloem RNA‐binding proteins as potential components of the long‐distance RNA transport system. Frontiers in Plant Science, 4, 130. 23675378 10.3389/fpls.2013.00130 PMC3650515

[mpp13022-bib-0071] Pallas, V. , Sanchez‐Navarro, J.A. , Kinard, G.R. & Di Serio, F. (2017) Molecular hybridization techniques for detecting and studying viroids In: Hadidi A., Randles J., Flores A. and Palukaitis P. (Eds.), Viroids and Satellites. Cambridge: Academic Press ‐ Elsevier Inc., pp. 369–379.

[mpp13022-bib-0072] Palukaitis, P. (1987) Potato spindle tuber viroid: investigation of the long‐distance, intra‐plant transport route. Virology, 158, 239–241. 18644563 10.1016/0042-6822(87)90260-1

[mpp13022-bib-0073] Papaefthimiou, I. , Hamilton, A. , Denti, M. , Baulcombe, D. , Tsagris, M. & Tabler, M. (2001) Replicating potato spindle tuber viroid RNA is accompanied by short RNA fragments that are characteristic of post‐transcriptional gene silencing. Nucleic Acids Research, 29, 2395–2400. 11376158 10.1093/nar/29.11.2395 PMC55696

[mpp13022-bib-0074] Qi, Y. & Ding, B. (2003) Differential subnuclear localization of RNA strands of opposite polarity derived from an autonomously replicating viroid. The Plant Cell, 15, 2566–2577. 14555700 10.1105/tpc.016576 PMC280561

[mpp13022-bib-0075] Radisek, S. , Majer, A. , Jakse, J. , Javornik, B. & Matoušek, J. (2012) First report of hop stunt viroid infecting hop in Slovenia. Plant Disease, 96, 592. 10.1094/PDIS-08-11-0640-PDN 30727422

[mpp13022-bib-0076] Reanwarakorn, K. & Semancik, J.S. (1998) Regulation of pathogenicity in hop stunt viroid‐related group II citrus viroids. Journal of General Virology, 79, 3163–3171. 10.1099/0022-1317-79-12-3163 9880036

[mpp13022-bib-0077] Reanwarakorn, K. & Semancik, J.S. (1999) Correlation of hop stunt viroid variants to cachexia and xyloporosis diseases of citrus. Phytopathology, 89, 568–574. 18944692 10.1094/PHYTO.1999.89.7.568

[mpp13022-bib-0078] Riesner, D. , Henco, K. , Rokohl, U. , Klotz, G. , Kleinschmidt, A.K. , Domdey, H. et al. (1979) Structure and structure formation of viroids. Journal of Molecular Biology, 133, 85–115. 529284 10.1016/0022-2836(79)90252-3

[mpp13022-bib-0079] Rodio, M.E. , Delgado, S. , de Stradis, A. , Gómez, M.D. , Flores, R. & Di Serio, F. (2007) A viroid RNA with a specific structural motif inhibits chloroplast development. The Plant Cell, 19, 3610–3626. 18055612 10.1105/tpc.106.049775 PMC2174877

[mpp13022-bib-0080] Sano, T. , Barba, M. , Li, S.F. & Hadidi, A. (2010) Viroids and RNA silencing: mechanism, role in viroid pathogenicity and development of viroid‐resistant plants. GM Crops, 1, 80–86. 21865875 10.4161/gmcr.1.2.11871

[mpp13022-bib-0081] Sano, T. , Hataya, T. , Terai, Y. & Shikata, E. (1989) Hop stunt viroid strains from dapple fruit disease of plum and peach in Japan. Journal of General Virology, 70, 1311–1319. 10.1099/0022-1317-70-6-1311 2732717

[mpp13022-bib-0082] Sano, T. , Mimura, R. & Ohshima, K. (2001) Phylogenetic analysis of hop and grapevine isolates of hop stunt viroid supports a grapevine origin for hop stunt disease. Virus Genes, 22, 53–59. 11210940 10.1023/a:1008182302704

[mpp13022-bib-0083] Sano, T. , Uyeda, I. , Shikata, E. , Ohno, T. & Okada, Y. (1984) Nucleotide sequence of cucumber pale fruit viroid: Homology to hop stunt viroid. Nucleic Acids Research, 12, 3427–3434. 6328420 10.1093/nar/12.8.3427 PMC318759

[mpp13022-bib-0084] Sasaki, M. & Shikata, E. (1977) On some properties of hop stunt disease agent, a viroid. Proceedings of the Japan Academy. Series B, Physical and Biological Sciences, 53, 109–112.

[mpp13022-bib-0085] Semancik, J.S. , Roistacher, C.N. , Rivera‐Bustamante, R. & Duran‐Vila, N. (1988) Citrus cachexia viroid, a new viroid of citrus: relationship to viroids of the exocortis disease complex. Journal of General Virology, 69, 3059–3068.

[mpp13022-bib-0086] Serra, P. , Gago, S. & Duran‐Vila, N. (2008) A single nucleotide change in Hop stunt viroid modulates citrus cachexia symptoms. Virus Research, 138, 130–134. 18789983 10.1016/j.virusres.2008.08.003

[mpp13022-bib-0087] Solovyev, A.G. , Makarova, S.S. , Remizowa, M.V. , Lim, H.S. , Hammond, J. , Owens, R.A. et al. (2013) Possible role of the Nt‐4/1 protein in macromolecular transport in vascular tissue. Plant Signaling and Behavior, 8, e25784. 10.4161/psb.25784 PMC4091084 23887490

[mpp13022-bib-0088] Steger, G. & Perreault, J.P. (2016) Structure and associated biological functions of viroids. Advances in Virus Research, 94, 141–172. 26997592 10.1016/bs.aivir.2015.11.002

[mpp13022-bib-0089] Steger, G. & Riesner, D. (2018) Viroid research and its significance for RNA technology and basic biochemistry. Nucleic Acids Research, 46, 10563–10576. 30304486 10.1093/nar/gky903 PMC6237808

[mpp13022-bib-0090] Su, X. , Fu, S. , Qian, Y. , Xu, Y. & Zhou, X. (2015) Identification of Hop stunt viroid infecting *Citrus limon* in China using small RNAs deep sequencing approach. Virology Journal, 12, 1–5. 26148502 10.1186/s12985-015-0332-2 PMC4492010

[mpp13022-bib-0091] Ueki, S. & Citovsky, V. (2001) RNA commutes to work: regulation of plant gene expression by systemically transported RNA molecules. BioEssays, 23, 1087–1090. 11746226 10.1002/bies.10027

[mpp13022-bib-0092] Vamenani, R. , Rahimian, H. , Alavi, S.M. , Pakdin Parizi, A. & Mirza Razzaz, T. (2019) Genetic diversity of hop stunt viroid from symptomatic and asymptomatic citrus trees in Iran. Journal of Phytopathology, 167, 484–489.

[mpp13022-bib-0094] Wang, M.B. , Bian, X.Y. , Wu, L.M. , Liu, L.‐X. , Smith, N.A. , Isenegger, D. et al. (2004) On the role of RNA silencing in the pathogenicity and evolution of viroids and viral satellites. Proceedings of the National Academy of Sciences of the United States of America, 101, 3275–3280. 14978267 10.1073/pnas.0400104101 PMC365780

[mpp13022-bib-0095] Wassenegger, M. , Heimes, S. , Riedel, L. & Sänger, H.L. (1994) RNA‐directed de novo methylation of genomic sequences in plants. Cell, 76, 567–576. 8313476 10.1016/0092-8674(94)90119-8

[mpp13022-bib-0096] Xia, C. , Li, S. , Hou, W. , Fan, Z. , Xiao, H. , Lu, M. et al. (2017) Global transcriptomic changes induced by infection of cucumber (*Cucumis sativus* L.) with mild and severe variants of hop stunt viroid. Frontiers in Microbiology, 8, 2427. 29312160 10.3389/fmicb.2017.02427 PMC5733102

[mpp13022-bib-0097] Xu, L. , Wang, J.W. , Zhu, D.Z. , Zong, X.J. , Wei, H.R. , Chen, X. et al. (2017) First report of hop stunt viroid from sweet cherry with dapple fruit symptoms in China. Plant Disease, 101, 394.

[mpp13022-bib-0098] Yang, X. , Hadidi, A. & Garnsey, S. (1992) Enzymatic cDNA amplification of citrus exocortis and cachexia viroids from infected citrus hosts. Phytopathology, 82, 279.

[mpp13022-bib-0103] Yang Y.‐A. , Wang H.‐Q. , Wu Z.‐J. , Cheng Z.‐M. , Li S.‐F. (2008) Molecular variability of hop stunt viroid: identification of a unique variant with a tandem 15‐nucleotide repeat from naturally infected plum tree. Biochemical Genetics, 46, 113–123. 18324471 10.1007/s10528-007-9134-6

[mpp13022-bib-0099] Zhang, Z. , Xia, C. , Matsuda, T. , Taneda, A. , Murosaki, F. , Hou, W. et al. (2020) Effects of host‐adaptive mutations on hop stunt viroid pathogenicity and small RNA biogenesis. International Journal of Molecular Sciences, 21, 7383. 10.3390/ijms21197383 PMC7582576 33036282

[mpp13022-bib-0100] Zhang, Z. , Zhou, Y. , Guo, R. , Mu, L. , Yang, Y. , Li, S. et al. (2012) Molecular characterization of Chinese hop stunt viroid isolates reveals a new phylogenetic group and possible cross transmission between grapevine and stone fruits. European Journal of Plant Pathology, 134, 217–225.

[mpp13022-bib-0101] Zhao, Y. , Owens, R.A. & Hammond, R.W. (2001) Use of a vector based on *Potato virus X* in a whole plant assay to demonstrate nuclear targeting of potato spindle tuber viroid. Journal of General Virology, 82, 1491–1497. 10.1099/0022-1317-82-6-1491 11369895

[mpp13022-bib-0102] Zhu, Y. , Qi, Y. , Xun, Y. , Owens, R. & Ding, B. (2002) Movement of potato spindle tuber viroid reveals regulatory points of phloem‐mediated RNA traffic. Plant Physiology, 130, 138–146. 12226494 10.1104/pp.006403 PMC166547

